# Implications of two-component systems *EnvZ*/*OmpR* and *BaeS*/*BaeR* in *in vitro* temocillin resistance in *Escherichia coli*

**DOI:** 10.1093/jac/dkae021

**Published:** 2024-02-02

**Authors:** Patricia Pérez-Palacios, José Luis Rodríguez-Ochoa, Ana Velázquez-Escudero, Jesús Rodríguez-Baño, José Manuel Rodríguez-Martínez, Álvaro Pascual, Fernando Docobo-Pérez

**Affiliations:** Unidad Clínica de Enfermedades Infecciosas y Microbiología, Hospital Universitario Virgen Macarena, Seville, Spain; Instituto de Biomedicina de Sevilla IBIS, Hospital Universitario Virgen Macarena/CSIC/Universidad de Sevilla, Sevilla, Spain; Unidad Clínica de Enfermedades Infecciosas y Microbiología, Hospital Universitario Virgen Macarena, Seville, Spain; Instituto de Biomedicina de Sevilla IBIS, Hospital Universitario Virgen Macarena/CSIC/Universidad de Sevilla, Sevilla, Spain; Unidad Clínica de Enfermedades Infecciosas y Microbiología, Hospital Universitario Virgen Macarena, Seville, Spain; Unidad Clínica de Enfermedades Infecciosas y Microbiología, Hospital Universitario Virgen Macarena, Seville, Spain; Instituto de Biomedicina de Sevilla IBIS, Hospital Universitario Virgen Macarena/CSIC/Universidad de Sevilla, Sevilla, Spain; Departamento de Medicina, Facultad de Medicina, Universidad de Sevilla, Sevilla, Spain; Centro de Investigación Biomédica en Red en Enfermedades Infecciosas (CIBERINFEC), Instituto de Salud Carlos III, Madrid, Spain; Instituto de Biomedicina de Sevilla IBIS, Hospital Universitario Virgen Macarena/CSIC/Universidad de Sevilla, Sevilla, Spain; Centro de Investigación Biomédica en Red en Enfermedades Infecciosas (CIBERINFEC), Instituto de Salud Carlos III, Madrid, Spain; Departamento de Microbiología, Facultad de Medicina, Universidad de Sevilla, Sevilla, Spain; Unidad Clínica de Enfermedades Infecciosas y Microbiología, Hospital Universitario Virgen Macarena, Seville, Spain; Instituto de Biomedicina de Sevilla IBIS, Hospital Universitario Virgen Macarena/CSIC/Universidad de Sevilla, Sevilla, Spain; Centro de Investigación Biomédica en Red en Enfermedades Infecciosas (CIBERINFEC), Instituto de Salud Carlos III, Madrid, Spain; Departamento de Microbiología, Facultad de Medicina, Universidad de Sevilla, Sevilla, Spain; Instituto de Biomedicina de Sevilla IBIS, Hospital Universitario Virgen Macarena/CSIC/Universidad de Sevilla, Sevilla, Spain; Centro de Investigación Biomédica en Red en Enfermedades Infecciosas (CIBERINFEC), Instituto de Salud Carlos III, Madrid, Spain; Departamento de Microbiología, Facultad de Medicina, Universidad de Sevilla, Sevilla, Spain

## Abstract

**Background:**

BaeS/BaeR is a two-component system of *Escherichia coli* that controls the expression of porins and efflux pumps. Its role in beta-lactam resistance is limited.

**Objectives:**

To study the role of *baeS/baeR* two-component system in temocillin resistance in *E. coli*.

**Methods:**

*E. coli* strain BW25113 and single-gene deletion mutants related to two-component systems were collected from the KEIO collection. Double-gen deletion mutants were generated. Temocillin-resistant mutant frequencies were determined at 32 mg/L. *E. coli* BW25113 mutants were selected by selective pressure from serial passages. Biological costs were analysed by growth curves. Genomes of the generated mutants were sequenced. The expression level of the *mdtA*, *mdtB*, *mdtC*, *acrD* and *tolC* in the Δ*baeS* mutant was determined by RT–PCR (with/without temocillin exposure).

**Results:**

The frequency of temocillin mutants ranged from 2.12 × 10^−8^ to 4.51 × 10^−8^ in single-porin mutants. No mutants were recovered from *E. coli* BW25113 (>10^−9^). Selection of temocillin-resistant variants by serial passage yielded mutants up to 128 mg/L. Mutations were found in the *baeS* gene. Temocillin MICs ranged from 4 to 32 mg/L (highest MICs for Δ*baeS* and Δ*ompR*). The efflux pumps *mdtA*, *mdtB*, *mdtC* and *acrD* pumps were overexpressed 3–10-fold in the presence of temocillin in Δ*baeS* compared to control.

**Conclusions:**

Mutations in the sensor histidine kinase, *baeS*, may be involved in temocillin resistance through the expression of the efflux pumps *mdtABC* and *acrD*. In addition, the low mutation rate may be a good predictor of temocillin activity.

## Introduction

Antimicrobial resistance is one of the biggest threats facing public health in infectious diseases. In recent years, the increase in multidrug-resistant *Enterobacterales* has been a challenge in patient management due to the reduction in therapeutic options. Despite the importance in promoting research and development of new antibiotics, investment from the pharmaceutical industry and biotech companies is declining and, in the last decade, the FDA has approved only 17 new systemic antibiotics.^1,2^ Owing to the lack of new antibiotic molecules, ‘old antibiotics’ such as colistin and some aminoglycosides have been rescued to treat infections caused by to multidrug-resistant bacteria.^3^ In this context, the use of temocillin, a semi-synthetic 6-α-methoxy derivative of ticarcillin marketed in the 1980s with a spectrum of action against *Enterobacterales* and currently available for use in countries such as Belgium, France, Germany, Luxembourg and the UK has been recovered.^4^

Resistance to β-lactams in Enterobacterales is mainly mediated by the production of ESBL, AmpC and carbapenemases.^5^ The temocillin stability against ESBL- and AmpC-type β-lactamases and some carbapenemases such as KPC-type, has made it an alternative to antibiotics of last resort such as carbapenems, avoiding its overuse.^6^ However, although the main β-lactam resistance mechanisms are mediated by enzyme production, changes in membrane permeability also play an important role through alterations in the expression of outer membrane porins (Omps) and/or efflux pumps, causing resistance in *Enterobacterales.*^7,8^ However, these types of study are scarce in temocillin, whose resistance mechanisms remain largely unknown.

Porins and efflux pumps are channels located in the plasma membrane of Gram-negative bacteria required for the exchange of small molecules and nutrients, whose expression is regulated by changes in external stress conditions.^9^ Some of these are tightly regulated by two-component systems, consisting of a membrane-bound histidine kinase that senses changes in the environment and sends a response to a regulator that modulates the differential expression of the genes involved.^10^ The e*nvZ/ompR* system is a two-component system in which *envZ* has the role of a histidine kinase sensor and *omp*R acts as a regulator of two porins, *ompC* and *ompF*. There are other porins, including *ompA*, *ompX* and *ompW*, but although their regulation is also modulated by environmental conditions, they appear to be independent of the control of the *envZ/ompR* system.^11^ While *ompA* plays an important role in maintaining the membrane integrity,^12^  *ompW* and *ompX* have been implicated in different responses to external stresses, including iron homeostasis.^13^ In addition to the *envZ/ompR*, another two-component system, *baeS/baeR*, has also been implicated in drug resistance in *E. coli.*^14^ The sensor kinase BaeS and the response regulator BaeR modulate the expression of inner efflux pumps *mdtABC* and *acrD*,^15^ which, in conjunction with the outer membrane efflux pump *tolC*, allow the efflux of a variety of compounds from the outer membrane.^16^ Moreover, recent studies suggest that single point mutations in the BaeS increase temocillin resistance in *Enterobacter cloacae* complex, although more studies are needed to confirm this in other species of Enterobacterales.^17^

Therefore, the objective of this study was to determine the role of porins and efflux pumps in resistance mechanisms to temocillin in *E. coli* under the control of the two-component systems *envZ*/*ompR* and *baeS*/*baeR*.

## Materials

### Bacterial strains

The wild-type *E. coli* BW25113 strain and single-gene deletion mutants related to two-component systems that express porins *envZ*/*ompR* (Δ*envZ*, Δ*ompR*, Δ*ompA*, Δ*ompC*, Δ*ompF*, Δ*ompW*, Δ*omprX*) and efflux pumps *baeS*/*baeR* (Δ*baeS*, Δ*baeR*, Δ*tolC*, Δ*acrD*, Δ*mdtA*, Δ*mdtB* and Δ*mdtC*), were collected from the KEIO collection.^18^ The double deletion mutants, Δ*ompR*-Δ*ompF*, Δ*ompR*-Δ*ompW*, Δ*ompR*-Δ*ompX*, Δ*ompR*-Δ*ompA*, Δ*ompC*-Δ*ompR*, Δ*ompC*-Δ*ompF*, Δ*ompW*-Δ*ompC*, Δ*ompX*-Δ*ompC*, Δ*ompA*-Δ*ompC*, Δ*ompF*-Δ*ompX* and Δ*ompX*-Δ*ompW*, were generated by phage P1vir transduction (Coli Genetic Stock Center, Yale University) from the previously selected strains, as described.^19^

### Temocillin mutant frequency determination

Temocillin-resistant mutant frequencies were evaluated for the wild-type *E. coli* BW25133 and the single-gene deletion porin mutants (*ΔompR*, Δ*ompF*, Δ*ompC*, Δ*ompA*, Δ*ompX* and Δ*ompW*). Mutant frequencies were determined in quadruplicate as previously described.^20^ Briefly, bacterial cultures were plated on drug-free MHA and MHA plates supplemented with 32 mg/L of temocillin (Eumedica, Belgium), colonies were enumerated after 24 h incubation at 37°C and mutant frequencies were calculated as the ratio of temocillin-resistant mutant cfu to total cfu.

### In vitro *selection of temocillin resistance via serial passages and bacterial fitness*

Selection of resistance to temocillin in *E. coli* BW25113 was performed by serial passage in 2-fold temocillin concentration in each subculture, in a range of 2–128 mg/L. Serial passage experiments were performed on MHA plates supplemented with each temocillin concentration. The fitness cost was measured by the growth time (doubling time in h) in *E. coli* BW25113, the mutants recovered at each temocillin concentration above 16 mg/L and the mutants obtained in temocillin frequency assay. Growth curves were generated in 96-well plates at 37°C and read every hour at OD_600_ (Infinite Nano 200 Pro; TECAN, Switzerland). Analyses were performed by fitting a non-parametric form (smoothing factor 0.55) the experimental growth curves using QurvE v.1.1 and the means values of each mutant were compared to control *E. coli* BW25113 by Student’s *t*-test (*P *± 0.05).^21^

### Whole-genome sequencing

The bacterial genomes of the mutants recovered from the serial passage study at concentrations >16 mg/L temocillin and the mutants obtained from the mutant frequency assay at 32 mg/L temocillin were sequenced using the MiSeq platform system (Illumina, San Diego, CA, USA). DNA extractions were performed with automatic system MagCore HF16 Plus (RCBBioscience, New Taipei City, Taiwan). Sample library was prepared with the Nextera XT DNA library preparation kit (Illumina, CA, USA) and DNA sequencing was carried out with the MiSeq Reagent Kit V3 (600 cycles) and the Illumina MiSeq sequencer (2 × 300 paired-end reads). The reads were quality filtered and *de novo* assembling of raw reads into contigs using were performed using CLC Genomic Workbench v.9.1 (Qiagen, Hilden, Germany). Mutations were called using the Breseq variant report v.0.35 via Galaxy/Pasteur (https://galaxy.pasteur.fr) using annotated genome of *E. coli* BW25113 as reference (NZ_CP009273.1).^22^

### Antimicrobial susceptibility testing

Antimicrobial susceptibility testing to temocillin (Eumedica, Belgium), ertapenem (Sigma Aldrich, USA), tobramycin (Sigma Aldrich), fosfomycin (Santa Cruz Biotechnology, USA) and ciprofloxacin (Sigma Aldrich, USA) was determined by microdilution assay according to ISO 20776-1:2019 (European Committee for Standardization 2006). Microdilution was performed in 96-well plates containing concentration between 128 and 0.06 mg/L in MHB medium for temocillin, tobramycin and fosfomycin and between 16 and 0.002 mg/L for ertapenem and ciprofloxacin. The MIC of each antibiotic was selected by the mean of three determinations considering as the lowest concentration showing no visible growth. To study possible cross-resistance for *E. coli* BW25113, Δ*baeS* mutant, mutants obtained after sequential passages and single-gene deletion porin mutants, disc diffusion was performed for ampicillin, cefuroxime, cefoxitin, cefotaxime, ceftazidime, cefepime, aztreonam, ertapenem, imipenem and meropenem in accordance with EUCAST guidelines. Also, heteroresistance phenotype to temocillin, done by gradient strip assays following the manufacturer’s instructions, to *E. coli* ATCC 25922 was used as a control strain.

### Real-time quantitative RT–PCR (RT–qPCR)

The genes encoding pumps regulated by the two-component *baeS/baeR* system (*mdtA*, *mdtB*, *mdtC* and *acrD*) and the not-directly related genes *tolC* and *acrA*, were selected to verify the expression level of these. Expression levels were measured by RT–qPCR (LifeCycler, Roche, Switzerland) in the Δ*baeS* mutant using *E. coli* BW25113 as control, with or without exposure to temocillin concentrations of 16 and 2 mg/L, respectively. Total RNA was extracted using QIAcubesQiagen (Venlo, the Netherlands), following the manufacturer’s instructions. cDNA was synthesized from total RNA samples using transcriptor first strand cDNA synthesis kit (Roche, Switzerland). Real-time PCR was performed in the LightCycler 480 Instrument II (Roche, Switzerland) with specific primers (Supplementary Table S1, available as Supplementary data at *JAC* Online). *gyrB* was chosen as a housekeeping gene. The relative expression levels of each gene were calculated using the 2^−△△^Ct method. Experiments were performed in triplicate.

## Results

### Temocillin-resistant mutant characterization

The temocillin mutant frequencies for the isogenic collection are shown in Table 1. Inactivation of porin genes resulted in the selection of mutants at 32 mg/L in Δ*ompR*, Δ*ompF*, Δ*ompC*, Δ*ompX*, Δ*ompW* (Δ*ompR*-32, Δ*ompF*-32, Δ*ompC*-32, Δ*ompX*-32, Δ*ompW*-32). No Δ*ompA-derived* mutants were obtained. Temocillin mutant frequencies in these isolates ranged from 2.12 × 10^−8^ to 4.51 × 10^−8^ (Table 1). Mutants recovered from mutant frequencies assay showed a MIC to temocillin between 64–128 mg/L (Table 1). However, when one-step mutant frequency assay at a concentration of 32 mg/L of temocillin was performed in *E. coli* BW251113, no mutants were recovered. After serial passages, *E. coli* BW25113 was able to grow in duplicate at each temocillin concentrations of 4, 8, 16, 32, 64 and 128 mg/L. All mutants recovered from the serial passage assay showed a 2–32-fold increase MIC to temocillin over control *E. coli* BW25113 (Table 1). Regarding fitness cost analyses, mutants recovered at 16, 32, 64 and 128 mg/L significantly increased their doubling time between 1.5- and 2.9-fold longer than *E. coli* BW25113, with mutant BW25113-64 and the BW25113-128 showing the longest doubling time. The mutants obtained in the temocillin frequency assay showed no significant difference in their doubling time compared to the control *E. coli* BW25113 (Table 1).

**Table 1:** . Temocillin MIC (mg/L), position and mutations found in *baeS* gene in *E. coli* BW25113 and isogenic collection generated by serial passage in increasing temocillin concentration and mutant obtained from porin deficiency isolates in the mutant frequency assay (32 mg/L), temocillin mutant frequency (percentage of reads) and fitness cost measured by duplication time (h). Bold indicates amino acid change and position

Strain name	MIC (mg/L)	Information of mutations in *baeS* gene					
		Position	Codon change	Frequency	*P* value	Temocillin mutant frequency (mean + SD)	Duplication time (h)
*E. coli* BW25113	4	—	—	—	—	>10^−9^	0.67 ± 0.15
BW25113-16	16	ND	ND	ND	ND	—	1.03 ± 0.22^a^
BW25113-32	32	2 156 843	**Q163E** (CAG→GAG)	96%	0.053	—	1.48 ± 0.93^a^
BW25113-64	64	2 156 843	**Q163E** (CAG→GAG)	100%	0.001	—	1.94 ± 1.29^a^
BW25113-128	128	2 156 843	**Q163E** (CAG→GAG)	98%	0.034	—	1.54 ± 0.92^a^
Δ*ompR*-32	64	2 156 856	**S167I** (AGC→ATC)	100%	0.001	4.51 × 10^−8 ^± 2.06 × 10^−8^	0.76 ± 0.18
Δ*ompC*-32	64	2 157 165	**V270G** (GTG→GGG)	100%	0.001	2.74 × 10^−8 ^± 1.07 × 10^−8^	0.72 ± 0.17
Δ*ompF*-32	128	2 156 822	**D156N** (GAT→AAT)	100%	0.001	3.77 × 10^−8 ^± 2.76 × 10^−8^	0.70 ± 0.19
Δ*ompX*-32	64	2,156,469-2,156,476	Δ6 bp	100%	0.001	2.94 × 10^−8 ^± 1.66 × 10^−8^	0.68 ± 0.17
Δ*ompW*-32	64	2 156 856	**S167I** (AGC→ATC)	100%	0.001	2.12 × 10^−8 ^± 1.44 × 10^−8^	0.65 ± 0.14

ND, not detected.

^a^ *P *≤ 0.05.

Mutations relative to *E. coli* BW25113 were compared using Breseq mutation prediction pipeline. Mutants generated at 32, 64 and 128 mg/L of temocillin, in the serial passages assay, contained a significant number of reads carrying the mutation Q163E (CAG→GAG) (*P *< 0.001) in the histidine kinase sensor *baeS* at position 2 156 843 in the chromosome, whereas this mutation was not found in the mutant generated at 16 mg/L (Table 1). Analysis of the mutations in the isolates obtained from the mutant frequency assay, also identified point mutations in *baeS*, specifically S167I (AGC→ATC) in the *ompR* and *ompW* mutants, V270G (GTG→GGG) in the *ompC* mutant, D156N (GAT→AAT) in the *ompF* mutant. In the *ompX* mutant, a 6 bp deletion was found between positions 2156469 and 2156476 (Table 1).

### Antimicrobial susceptibility

The analysis of a *E. coli* BW25113 mutant isogenic collection allowed to us determine the impact of multiple porins and efflux pumps in temocillin resistance. MICs to temocillin ranged from 1 to 32 mg/L, with the highest MICs Δ*baeS* (MIC 32 mg/L) and Δ*ompR* (MIC 16 mg/L) mutants with an increase of 8- and 4-fold increase, respectively, compared to *E. coli* BW25113 (MIC of 4 mg/L) (Table 2). The lowest MIC was recorded in Δ*tolC* (MIC of 1 mg/L) with a 4-fold decrease in temocillin concentration compared to *E. coli* BW25113. Other antibiotics whose resistance is associated with changes in membrane permeability with loss of porins (ertapenem), efflux pumps (ciprofloxacin and tobramycin) or defects in the synthesis of transporters (fosfomycin), were also tested in the isogenic collection. For ertapenem, the MICs ranged from 0.003 to 0.5 mg/L. Of the single-gene deletion mutants, the loss of the *ompF* and *ompR* genes resulted in a 4- and 64-fold increase, in MIC, respectively. MICs to ciprofloxacin, fosfomycin and tobramycin in these mutants ranged from 0.001–0.125 mg/L, 1–4 and 0.25–2 mg/L, respectively, in these mutants. The *tolC* mutant showed a 2-fold decreased in MICs to these antimicrobials compared to *E. coli* BW25113. The temocillin MICs of the double-porin mutants ranged from 4 to 16 mg/L. Mutants with deletions in *ompR* reached an MIC at temocillin of 16 mg/L, with no differences with respect to the single *ompR* mutant. The double loss of the *ompC* and *ompF* porins did not increase the MIC at temocillin with respect to the *E. coli* BW25113 control. With respect to other antibiotics, double-porin mutants (except double-gene mutants of *ompA* or *ompW*) increased their MIC to ertapenem 4- to 64-fold compared to the control. There were no significant MIC changes to ciprofloxacin or tobramycin in these double-gene mutants. With respect to cross-resistance, a reduction in the size of ampicillin inhibition halo (3–4 mm, changing clinical category from susceptible to resistance) was observed in the mutants obtained with serial passages and a reduction in aztreonam inhibition halo (4 mm with no changes in clinical category) was observed for the BW-64 and BW-128 mutants. No cross-resistance was found for the rest of the β-lactams studied (Supplementary Table S2). Regarding heteroresistance phenotype, colonies within the ampicillin inhibition halo were observed in the strains studied, including for the control *E. coli* BW-25113 (Supplementary Table S2). Colonies or double inhibition halos were also observed near the ellipse of the temocillin gradient strips for all the strains studied (Supplementary Figure S1).

**Table 2.** Antimicrobial susceptibility (MIC, mg/L) of *E. coli* BW25113 mutant isogenic collection

Strain name	MIC (mg/L)				
	TEM	ERT	CIP	FOT	TOB
ATCC25922	16	0.008	0.008	1	0.5
BW25113	4	0.008	0.008	1	0.5
Δ*envZ*	8	0.004	0.008	1	1
Δ*ompR*	16	0.5	0.016	4	1
Δ*ompA*	8	0.004	0.008	2	1
Δ*ompC*	4	0.004	0.008	2	0.5
Δ*ompF*	16	0.06	0.016	2	2
Δ*ompX*	8	0.016	0.008	1	1
Δ*ompW*	8	0.004	0.002	1	2
Δ*baeS*	32	0.006	0.008	1	1
Δ*baeR*	8	0.004	0.008	4	2
Δ*acrD*	8	0.004	0.125	1	1
Δ*mdtA*	8	0.016	0.125	2	0.5
Δ*mdtB*	8	0.016	0.004	2	0.5
Δ*mdtC*	8	0.016	0.004	1	0.5
Δ*tolC*	1	0.004	0.002	1	0.25
Δ*ompR*-Δ*ompC*	16	0.25	0.006	8	0.5
Δ*ompR*-Δ*ompF*	16	0.5	0.016	8	0.5
Δ*ompR*-Δ*ompA*	8	0.125	0.016	2	0.25
Δ*ompR*-Δ*ompW*	16	0.125	0.016	4	0.5
Δ*ompC*-Δ*ompF*	8	0.5	0.016	8	0.5
Δ*ompC*-Δ*ompA*	4	0.016	0.006	2	0.25
Δ*ompC*-Δ*ompW*	4	0.06	0.006	2	0.5
Δ*ompC*-Δ*ompX*	4	0.006	0.006	1	0.5
Δ*ompF*-Δ*ompX*	16	0.03	0.03	2	0.5
Δ*ompF*-Δ*ompW*	8	0.03	0.03	32	1
Δ*ompA*-Δ*ompX*	8	0.016	0.016	4	0.5
Δ*ompA*-Δ*ompW*	4	0.016	0.016	1	1
Δ*ompW*-Δ*ompX*	4	0.016	0.016	2	1

### Efflux pumps expression

**Figure 1.**
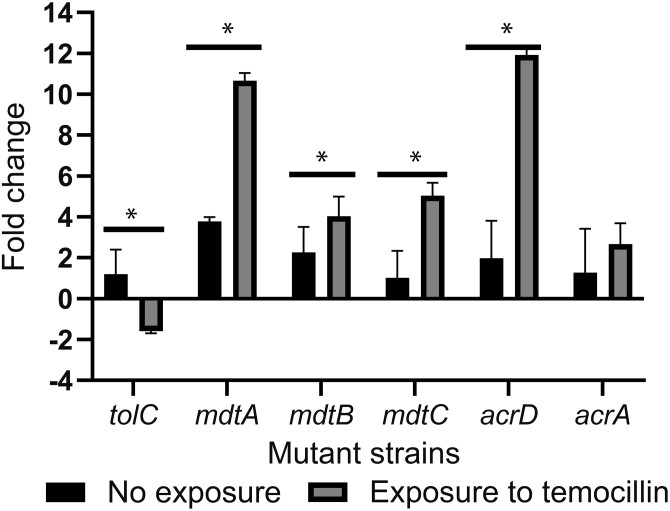
Fold change expression of the efflux pump components *tolC*, *mdtA*, *mdtB*, *mdtC*, *acrD* and *acrA* in the Δ*baeS* compared to *E. coli* BW25113 with and without temocillin exposure.

The genes encoding efflux pumps, regulated by the two-component *baeS/baeR* system in addition to *tolC* and *acrA*, were measure in response to temocillin in the Δ*baeS* mutant compared to the control *E. coli* BW25113. Without temocillin exposure, the expression of the studied efflux pumps did not change in the Δ*baeS* mutant compared to the control *E. coli* BW25113. When single mutant Δ*baeS* and *E. coli* BW25113 were exposed to 1/2xMIC of temocillin, *mdtA*, *mdtC* and *acrD* genes were overexpressed by 7-, 4- and 10-fold, respectively, in Δ*baeS* compared to the expression of these efflux pumps in the control *E. coli* BW25113. The expression levels of *tolC* and *acrA*, did not change on exposure to temocillin (Figure 1).

## Discussion

This work provides insight into the resistance mechanisms to temocillin in *E. coli* through the involvement of the two-component systems *envZ*/*ompR* and *baeS*/*baeR*, finding that mutations or deletions in the sensor histidine kinase *BaeS* lead to an increase in MIC to temocillin, mediated by overexpression in the efflux pumps *acrD*, *mdtABC*.

Temocillin is a recently rescued forgotten antibiotic due to its resistance to hydrolysis by Ambler class A and C β-lactamases.^6^ However, the molecular basis of resistance to temocillin remains largely unknown and although alterations in membrane permeability and the enzymatic barrier are known to contribute to antibiotic resistance in *E. coli*,^12,23^ there are few studies elucidating it with temocillin.

It is proposed that the frequency of selection of mutants *in vitro* to temocillin is low, as is the selection of resistant mutants detected *in vivo.*^24^ In terms of the frequency of spontaneous mutant appearance, the frequency of appearance of single-porin mutants was 10^−8^, in contrast to the results obtained with *E. coli* BW25113 where no temocillin-resistant mutant was recovered. These data agree with other studies where the frequency of occurrence of temocillin mutants reached 10^−8^.^25^ However, mutants were recovered from *E. coli* BW25113 during serial passage. In all these recovered mutants, the MIC to temocillin was increased between 2- and 32-fold, compared to control *E. coli* BW25113. Analysis of the relative mutations in mutants obtained at 32, 64 and 128 mg/L of temocillin, showed various changes in the BaeS histidine kinase sensor as well as in the porin mutants recovered from the mutant frequency assay. In this work, the doubling time of the mutants recovered in the serial passaging assay with temocillin was slightly longer than that of the control *E. coli* BW25113. It is known that strains with mutations conferring antibiotic resistance often have a lower growth rate, but these mutations may confer a survival advantage that selects for the resistant population.^26^ Although temocillin resistance was not reversed and the MIC to temocillin remained stable in the mutants, it is unclear how this fitness cost could impact clinical isolates or if this biological cost could be compensated for by other mutations.

The regulation of the membrane permeability through reducing uptake by downregulating the expression of porins, or by increasing the efflux of compounds by overexpressing efflux pumps, is mediated by two-component systems.^27^ The two-component systems, Envz/OmpR and BaeS/BaeR, regulate membrane permeability via porins and efflux pumps, respectively. In this study, the MIC to temocillin was studied in single mutants of several porins in an isogenic collection. Although it was originally thought that resistance to temocillin could be due to a reduced entry of the molecule into the cell through the porins of the outer membranes,^28^ single mutants of Δ*ompC*, Δ*ompF*, Δ*ompA*, Δ*ompX* and Δ*ompW* did not show a significant increase in their MIC to temocillin compared to the control *E. coli* BW25113. An increase in 4xCMI was only observed for the single -mutant Δ*ompR*, the main regulator of OmpC and *OmpF*, porins for which changes in their expression have been described to confer a decreased susceptibility to some beta-lactams such as ertapenem.^29^ Indeed, loss of the *ompF* and *ompR* genes resulted in 4- and 64-fold increases in MIC to ertapenem, respectively. Whereas there are studies linking resistance to other beta-lactams, such as ceftriaxone, in *S. enterica*, mediated by OmpW and OmpX-like porins,^30^ no increase in MIC to temocillin was observed for the loss of these in our study. Similarly, no changes in MICs to temocillin were observed in the double-porin mutants, which obtained the same MIC values as their single mutants.

The BaeS/BaeR two-component system has also been linked to antimicrobial resistance in *E. coli*.^31^ Moreover, it has been described that overproduction of BaeR can also cause novobiocin resistance through overexpression of the drug efflux system *mdtABC*.^32^ In this work, mutation and deletions cause overexpression of the efflux pump *mdtABC*, and especially of *acrD*. Moreover, Δ*baeS* mutant increased its MIC to temocillin up to 8-fold in compared to the control. In addition to novobiocin resistance, increased erythromycin resistance has also been described by gain-of-function mutations in *baeS* that constitutively activate the *baeSR* two-component regulatory system to increase expression of the efflux pump *mdtABC*.^33^ Although in Enterobacterales both *baeS* and *baeR* have been implicated in resistance to ciprofloxacin through activation of the histidine kinase sensor^34^ and to ceftriaxone through loss of *baeR*,^30^ the involvement of the *baeS/baeR* two-component system in antibiotic resistance remains unclear. Recently, Guérin *et al.* showed that temocillin resistance in the *Enterobacter asburiae* can result from a single BaeS alteration, probably resulting in the permanent phosphorylation of BaeR and leading to AcrD overexpression and temocillin resistance through enhanced active efflux.^17^ However, because of the presence of polymorphisms present in *baeS* from different Enterobacterales species and even within the same species, it is not possible to compare these point-single mutations.

This work has certain limitations. This study used the KEIO collection, which is derived from a non-uropathogenic intestinal strain of *E. coli* K12. It is unclear how the selected mutations may affect other *E. coli* strains from different sources with varying responses to factors such as nutrient intake and cellular homeostasis, which are involved in the regulation of resistance mechanisms such as porins and efflux pumps. Also, this study was performed entirely under *in vitro* conditions and it is unknown what effects of two-component systems may affect temocillin resistance *in vivo*. Further studies are needed to clarify the *in vivo* implications of this temocillin resistance.

In conclusion, alterations in *E. coli* membrane porins may not be involved in temocillin resistance. However, this work has shown that mutations in the histidine kinase sensor *BaeS* can alter the expression of plasma membrane efflux pumps in *E. coli* by overexpressing the efflux pumps *mdtABC* and *acrD* contributing to temocillin resistance. Although this resistance has some biological cost in terms of growth rate, it does not appear to contribute significantly to the development of the mutants. In addition, the low rate of mutant emergence may be a good predictor of temocillin activity.

## Supplementary Material

dkae021_Supplementary_Data

## Supplementary data

Figure S1 and Tables S1 and S2 are available as Supplementary data at *JAC* Online.

## References

[1] Outterson K, Rex JH. Evaluating for-profit public benefit corporations as an additional structure for antibiotic development and commercialization. Transl Res 2020; 220: 182–90. 10.1016/j.trsl.2020.02.006 32165059

[2] Chahine EB, Dougherty JA, Thornby KA et al Antibiotic approvals in the last decade: are we keeping up with resistance? Ann Pharmacother 2022; 56: 441–62. 10.1177/10600280211031390 34259076

[3] Peri AM, Doi Y, Potoski BA et al Antimicrobial treatment challenges in the era of carbapenem resistance. Diagn Microbiol Infect Dis 2019; 94: 413–25. 10.1016/j.diagmicrobio.2019.01.020 30905487

[4] Balakrishnan I, Koumaki V, Tsakris A. Temocillin: is this the right momentum for its global use? Future Microbiol 2019; 14: 81–3. 10.2217/fmb-2018-0316 30644315

[5] Palzkill T . Structural and mechanistic basis for extended-spectrum drug-resistance mutations in altering the specificity of TEM, CTX-M, and KPC β-lactamases. Front Mol Biosci 2018; 5: 16. 10.3389/fmolb.2018.00016 29527530 PMC5829062

[6] Livermore DM, Tulkens PM. Temocillin revived. J Antimicrob Chemother 2009; 63: 243–5. 10.1093/jac/dkn511 19095679

[7] Blair JM, Webber MA, Baylay AJ et al Molecular mechanisms of antibiotic resistance. Nat Rev Microbiol 2015; 13: 42–51. 10.1038/nrmicro3380 25435309

[8] Abushaheen MA, Muzaheed, Fatani AJ et al Antimicrobial resistance, mechanisms and its clinical significance. Dis Mon 2020; 66: 100971. 10.1016/j.disamonth.2020.100971 32201008

[9] Masi M, Winterhalter M, Pagès JM. Outer membrane porins. Subcell Biochem 2019; 92: 79–123. 10.1007/978-3-030-18768-2_4 31214985

[10] Lingzhi L, Haojie G, Dan G et al The role of two-component regulatory system in β-lactam antibiotics resistance. Microbiol Res 2018; 215: 126–9. 10.1016/j.micres.2018.07.005 30172298

[11] Nakashima K, Horikoshi K, Mizuno T. Effect of hydrostatic pressure on the synthesis of outer membrane proteins in *Escherichia coli*. Biosci Biotechnol Biochem 1995; 59: 130–2. 10.1271/bbb.59.130 7765962

[12] Choi U, Lee CR. Distinct roles of outer membrane porins in antibiotic resistance and membrane integrity in *Escherichia coli*. Front Microbiol 2019; 10: 953. 10.3389/fmicb.2019.00953 31114568 PMC6503746

[13] Lin X, Wu L, Li H et al Downregulation of *Tsx* and *OmpW* and upregulation of *OmpX* are required for iron homeostasis in *Escherichia coli*. J Proteome Res 2008; 7: 1235–43. 10.1021/pr7005928 18220334

[14] Hirakawa H, Nishino K, Hirata T et al Comprehensive studies of drug resistance mediated by overexpression of response regulators of two-component signal transduction systems in *Escherichia coli*. J Bacteriol 2003; 185: 1851–6. 10.1128/JB.185.6.1851-1856.2003 12618449 PMC150137

[15] Leblanc SK, Oates CW, Raivio TL. Characterization of the induction and cellular role of the BaeSR two-component envelope stress response of *Escherichia coli*. J Bacteriol 2011; 193: 3367–75. 10.1128/JB.01534-10 21515766 PMC3133272

[16] Rosner JL, Martin RG. Reduction of cellular stress by TolC-dependent efflux pumps in *Escherichia coli* indicated by BaeSR and CpxARP activation of spy in efflux mutants. J Bacteriol 2013; 195: 1042–50. 10.1128/JB.01996-12 23264577 PMC3571319

[17] Guérin F, Gravey F, Reissier S et al Temocillin resistance in *the Enterobacter cloacae complex* is conferred by a single point mutation in BaeS, leading to overexpression of the AcrD efflux pump. Antimicrob Agents Chemother 2023; 67: e00358-23. 10.1128/aac.00358-23 37195180 PMC10269110

[18] Baba T, Ara T, Hasegawa M et al Construction of *Escherichia coli* K-12 in-frame, single-gene knockout mutants: the Keio collection. Mol Syst Biol 2006; 2: 2006.0008. 10.1038/msb4100050 PMC1681482 16738554

[19] Thomason LC, Costantino N, Court DL. *E. coli* genome manipulation by P1 transduction. Curr Protoc Mol Biol 2007; 79: 1.17.1–.8. 10.1002/0471142727.mb0117s79 18265391

[20] Ballestero-Téllez M, Docobo-Pérez F, Portillo-Calderón I et al Molecular insights into fosfomycin resistance in *Escherichia coli*. J Antimicrob Chemother 2017; 72: 1303–9. 10.1093/jac/dkw573 28093485

[21] Wirth NT, Funk J, Donati S et al Qurve: user-friendly software for the analysis of biological growth and fluorescence data. Nat Protoc 2023; 18: 2401–3. 10.1038/s41596-023-00850-7 37380826

[22] Deatherage DE, Barrick JE. Identification of mutations in laboratory-evolved microbes from next-generation sequencing data using Breseq. Methods Mol Biol 2014; 1151: 165–88. 10.1007/978-1-4939-0554-6_12 24838886 PMC4239701

[23] Webber MA, Piddock LJ. The importance of efflux pumps in bacterial antibiotic resistance. J Antimicrob Chemother 2003; 51: 9–11. 10.1093/jac/dkg050 12493781

[24] Alexandre K, Chau F, Guérin F et al Activity of temocillin in a lethal murine model of infection of intra-abdominal origin due to KPC-producing *Escherichia coli*. J Antimicrob Chemother 2016; 71: 1899–904. 10.1093/jac/dkw066 27029848

[25] Soubirou JF, Rossi B, Couffignal C et al Activity of temocillin in a murine model of urinary tract infection due to *Escherichia coli* producing or not producing the ESBL CTX-M-15. J Antimicrob Chemother 2015; 70: 1466–72. 10.1093/jac/dku542 25564564

[26] Durão P, Balbontín R, Gordo I. Evolutionary mechanisms shaping the maintenance of antibiotic resistance. Trends Microbiol 2018; 26: 677–91. 10.1016/j.tim.2018.01.005 29439838

[27] Ferrand A, Vergalli J, Pagès JM et al An intertwined network of regulation controls membrane permeability including drug influx and efflux in *Enterobacteriaceae*. Microorganisms 2020; 8: 833. 10.3390/microorganisms8060833 32492979 PMC7355843

[28] Verbist L . In vitro activity of temocillin (BRL 17421), a novel beta-lactamase-stable penicillin. Antimicrob Agents Chemother 1982; 22: 157–61. 10.1128/AAC.22.1.157 6982023 PMC183693

[29] Lartigue MF, Poirel L, Poyart C et al Ertapenem resistance of *Escherichia coli*. Emerg Infect Dis 2007; 13: 315–7. 10.3201/eid1302.060747 17479901 PMC2725854

[30] Hu WS, Li PC, Cheng CY. Correlation between ceftriaxone resistance of *Salmonella enterica* serovar Typhimurium and expression of outer membrane proteins OmpW and Ail/OmpX-like protein, which are regulated by BaeR of a two-component system. Antimicrob Agents Chemother 2005; 49: 3955–8. 10.1128/AAC.49.9.3955-3958.2005 16127081 PMC1195446

[31] Guest RL, Raivio TL. Role of the gram-negative envelope stress response in the presence of antimicrobial agents. Trends Microbiol 2016; 24: 377–90. 10.1016/j.tim.2016.03.001 27068053

[32] Nagakubo S, Nishino K, Hirata T et al The putative response regulator BaeR stimulates multidrug resistance of *Escherichia coli* via a novel multidrug exporter system, *MdtABC*. J Bacteriol 2002; 184: 4161–7. 10.1128/JB.184.15.4161-4167.2002 12107133 PMC135206

[33] Cho H, Misra R. Mutational activation of antibiotic-resistant mechanisms in the absence of major drug efflux systems of *Escherichia coli*. J Bacteriol 2021; 203: e0010921. 10.1128/JB.00109-21 33972351 PMC8223954

[34] Guerrero P, Collao B, Morales EH et al Characterization of the BaeSR two-component system from *Salmonella* Typhimurium and its role in ciprofloxacin-induced *mdtA* expression. Arch Microbiol 2012; 194: 453–60. 10.1007/s00203-011-0779-5 22173828

