# Supplementary material for: Implications of two-component systems EnvZ/OmpR and BaeS/BaeR in in vitro temocillin resistance in Escherichia coli
Source: J Antimicrob Chemother. 2024 Feb 2;79(3):641–7. doi: 10.1093/jac/dkae021 (PMC10904727; doi:10.1093/jac/dkae021)
Supplement: dkae021_Supplementary_Data [file dkae021_supplementary_data.zip › Supplementary Tables S1 and S2.docx]

Table S1: Primers used in real-time quantitative real time polymerase chain reaction (qRT-PCR).

| Gene | Primers | Annealing temperature (ºC) |
| --- | --- | --- |
| *mdtA* | F: AGGTCAAAGCAGGCGATTTA R: TGAAACCGAAGGCACCATTA | 60 |
| *mdtB* | F: CTGCAAACGGCGGTAGATAAA  R: CCTGCAAGGTAAACTGGTACTG | 60 |
| *mdtC* | F: CGATCCCGCTGTCGTATTT  R: CGATCCCGCTGTCGTATTT | 60 |
| *acrD* | F: CTACCGATGGTTCGATGGATAAA  R: CAGCCAGATACGCATGGAATA | 60 |
| *acrA* | F: TCACCAGTGACGGCATTAAG  R: ATAGCGCGTAGGGTGATAGA | 60 |
| *tolC* | F: GCAATATGGGCCAGAACAAAG  R: CCTGTTTCACCTGCGAGTTA | 60 |
| *gyrB* | F: GTACAGGATGACGGGCGCGG  R: GTGCAGACCGCCGGACACT | 60 |

| **Disc diameter (mm)** | | | | | | | | | | |
| --- | --- | --- | --- | --- | --- | --- | --- | --- | --- | --- |
| **Strain** | **AMP** | **CFM** | **FOX** | **CTX** | **CAZ** | **FEP** | **ATM** | **ERT** | **IMP** | **MER** |
| ***E. coli* BW25113** | 15 | 30 | 30 | 35 | 33 | 38 | 35 | 38 | 32 | 37 |
| **Δ*baeS*** | 12* | 27 | 29 | 35 | 31 | 39 | 35 | 37 | 32 | 37 |
| **BW25113-16** | 12* | 25 | 26 | 32 | 29 | 36 | 31 | 38 | 33 | 39 |
| **BW25113-32** | 11* | 28 | 27 | 33 | 30 | 38 | 32 | 40 | 29 | 39 |
| **BW25113-64** | 11* | 27 | 26 | 34 | 34 | 36 | 26 | 35 | 33 | 38 |
| **BW25113-128** | 11* | 29 | 26 | 34 | 28 | 39 | 26 | 37 | 35 | 39 |
| **∆*ompR*-32** | 15* | 33 | 30 | 36 | 36 | 40 | 33 | 39 | 34 | 38 |
| **∆*ompC*-32** | 14* | 29 | 27 | 35 | 30 | 39 | 30 | 38 | 33 | 38 |
| **∆*ompF*-32** | 14* | 30 | 26 | 36 | 30 | 40 | 30 | 37 | 34 | 38 |
| **∆*ompW*-32** | 14* | 32 | 27 | 36 | 30 | 40 | 30 | 39 | 33 | 38 |
| **∆*ompX*-32** | 14* | 30 | 29 | 36 | 28 | 40 | 31 | 37 | 33 | 38 |

Table S2. Inhibition halo diameter (mm) for ampicillin (AMP), cefuroxime (CFM), cefoxitin (FOX), cefotaxime (CTX), ceftazidime (CAZ), cefepime (FEP), aztreonam (AZT), ertapenem (ERT), imipenem (IMP) and meropenem (MER).

* Colonies observed inside the inhibition halo.
