# Supplementary figures and images for: Implications of two-component systems EnvZ/OmpR and BaeS/BaeR in in vitro temocillin resistance in Escherichia coli
Source: J Antimicrob Chemother. 2024 Feb 2;79(3):641–7. doi: 10.1093/jac/dkae021 (PMC10904727; doi:10.1093/jac/dkae021)

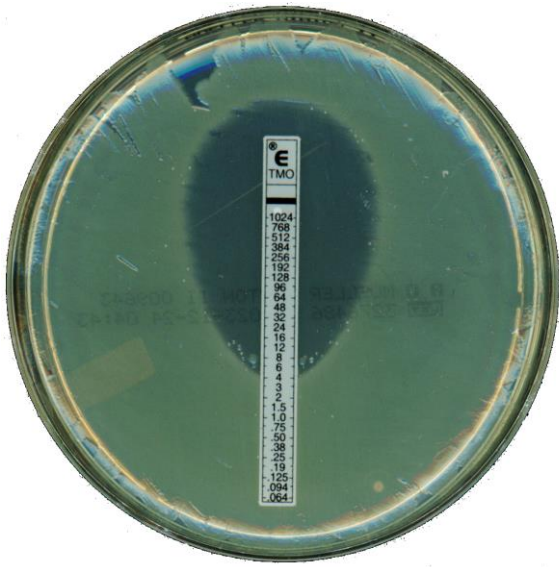

*E. coli* BW25113

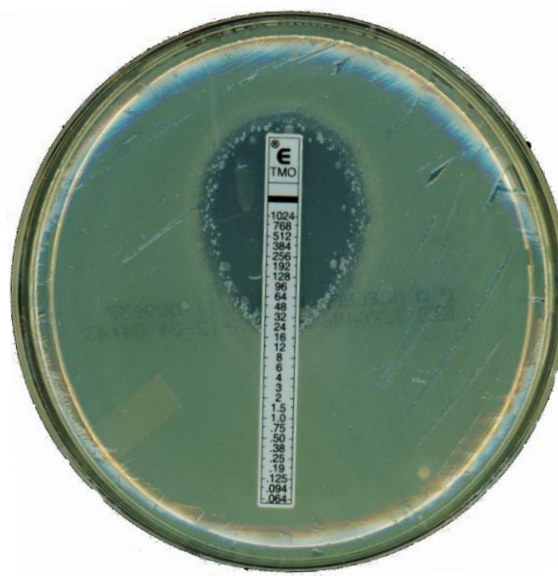

$\Delta baeS$

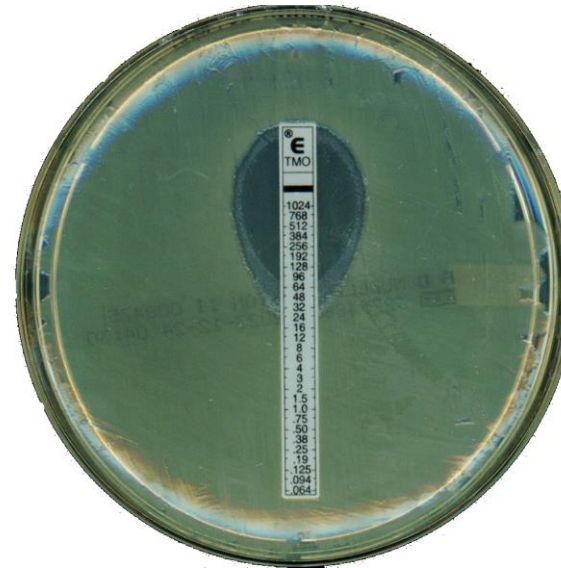

BW25113-16

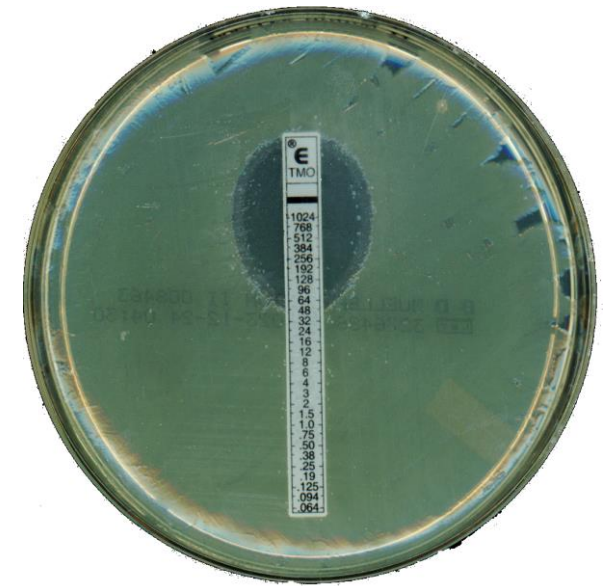

BW25113-32

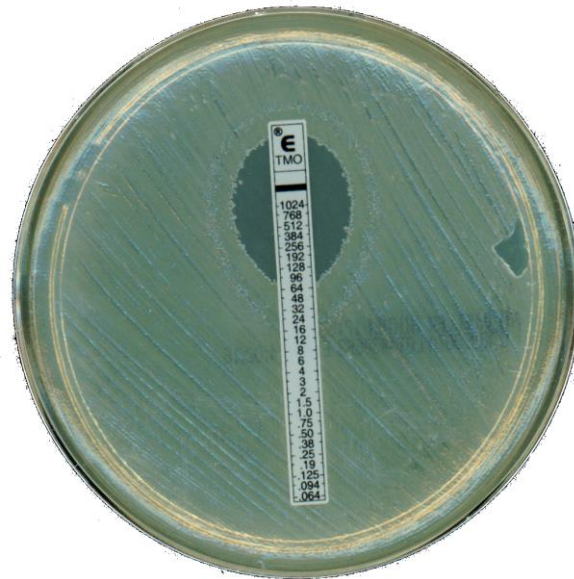

BW25113-64

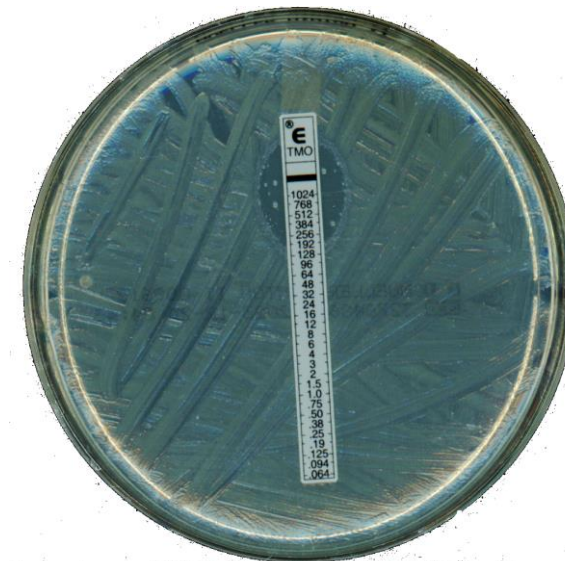

BW25113-128

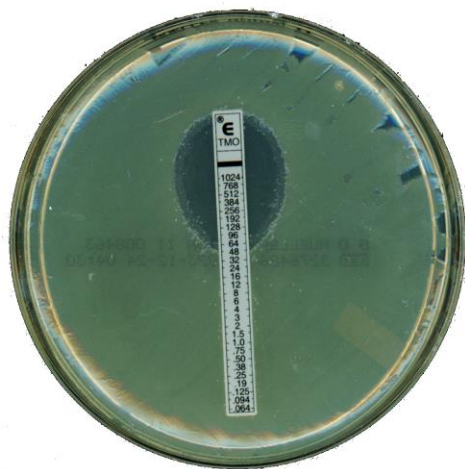

$\Delta ompR$ -32

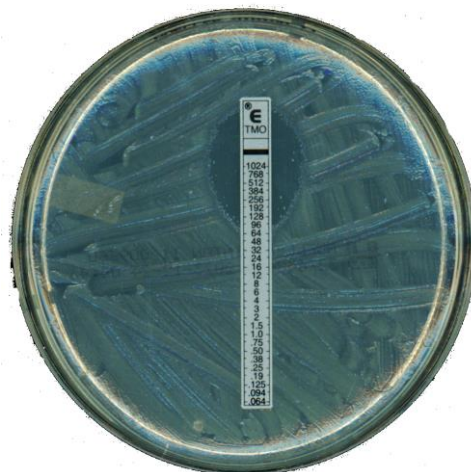

$\Delta ompC$ -32

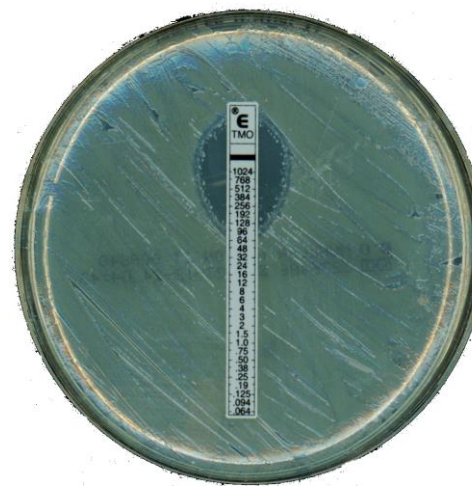

$\Delta ompF$ -32

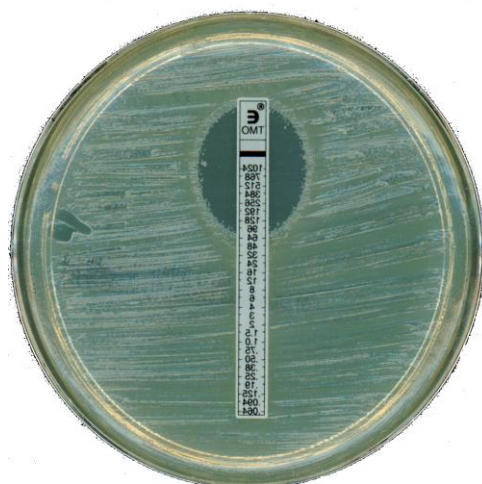

$\Delta ompW$ -32

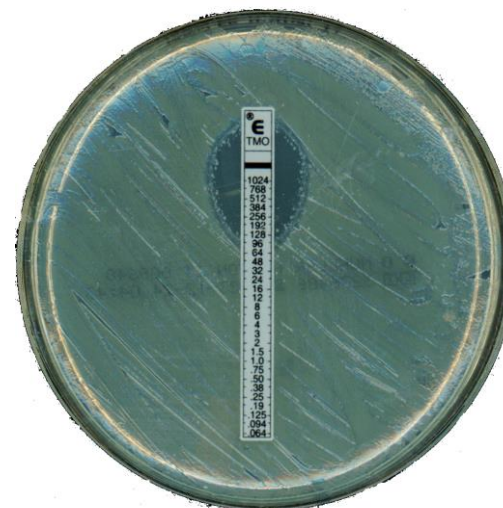

$\Delta ompW$ -32

Supplement: dkae021_Supplementary_Data [file dkae021_supplementary_data.zip › Supplementary Figure S1.pdf]
